# Imaging Synaptic Vesicle Protein SV2C with ^18^F‑UCB-F: An In Vitro Autoradiography and In Vivo NHP PET Study

**DOI:** 10.1021/acschemneuro.6c00241

**Published:** 2026-06-26

**Authors:** S. Nag, V. C. Sousa, R. Zou, A. F. Morén, P. Datta, Y. Khani, A. Valade, C. Vermeiren, P. Motte, J. Mercier, H. Ågren, C. Halldin, A. Varrone

**Affiliations:** † Department of Clinical Neuroscience, Karolinska Institutet, Stockholm 17176, Sweden; ‡ Department of Physics and Astronomy, 8097 Uppsala University, Uppsala 75120, Sweden; § UCB Biopharma, BE Research, Braine l’Alleud 1420, Belgium; ∥ HUN-REN TKI, Department of Biophysics and Radiation Biology, Semmelweis University, 1094 Budapest, Hungary

**Keywords:** PET, SV2C, radioligands, NHP, Fluorine-18, kinetics

## Abstract

The synaptic vesicle protein SV2C, predominantly found in the basal ganglia, has been associated with Parkinson’s disease through genetic studies. It plays a crucial role in regulating dopamine release and has been shown to be disrupted in PD animal models and brain tissues from PD patients. In the context of PD-related synaptopathy, SV2C may serve as a potential imaging target for monitoring disease progression and response to treatment. [^18^F]­UCB-F is a radioligand binding to SV2C developed by UCB. Preliminary autoradiography and PET studies in rats showed that [^18^F]­UCB-F displays a brain distribution consistent with the expression of SV2C *in vitro* but does not display any specific binding *in vivo*. This study was therefore designed to further investigate the affinity and selectivity of [^18^F]­UCB-F for SV2C and to examine the *in vitro* and *in vivo* properties of the radioligand in nonhuman primates. *In vitro* binding studies were performed to measure the affinity of UCB-F to SV2A, SV2B, and SV2C. In-silico modeling was used to assess the binding mode and energy of UCB-F. Autoradiography studies on rat and nonhuman primate (NHP) brain tissues were performed to confirm that [^18^F]­UCB-F showed similar distribution in rat and NHP tissue. Finally, PET studied in NHPs were performed to examine the *in vivo* pharmacokinetic properties of [^18^F]­UCB-F. [^18^F]­UCB-F was successfully synthesized from the corresponding precursor with high yield. Autoradiography on brain slices from rats and NHPs demonstrated specific binding of [^18^F]­UCB-F in the pallidum, striatum, substantia nigra, and brainstem, consistent with the known brain expression of SV2C. In NHPs, [^18^F]­UCB-F rapidly crossed the blood-brain barrier, reaching peak uptake values of 2.8%ID in NHP1 and 2.1%ID in NHP2 at 4 min postinjection. The tracer was rapidly washed out from the brain, with no clear regional distribution. Radiometabolite analysis revealed the formation of only more polar radiometabolites, with approximately 15% of unchanged radioligand remaining in plasma at 15 min postinjection. *In vitro* and *in-silico* studies demonstrated that the affinity of [^18^F]­UCB-F decreased by approximately one factor of magnitude with increase of temperature from 277 K (4 °C) to 310 K (37 °C). This temperature-related decrease of the affinity for SV2C together with rapid *in vivo* radiometabolism might explain the discrepancy between *in vitro* and *in vivo* performance of [^18^F]­UCB-F. Overall, these findings suggest that [^18^F]­UCB-F is not a suitable PET radioligand for imaging SV2C. Further research is needed to identify alternative candidates with improved in vivo stability and brain retention.

## Graphical abstract

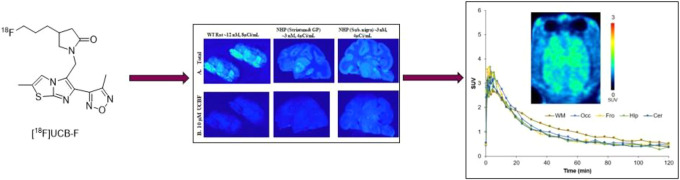

## Introduction

Synaptic vesicle glycoprotein 2C (SV2C) is a synaptic vesicle membrane protein in the SV2 family involved in vesicle trafficking and vesicle-filled neurotransmitter release.^1^ The three paralogs SV2A, SV2B, and SV2C show differences in the regional brain expression in the brain and diverse functions.^2,3^ In contrast to the broadly located SV2A, SV2C has a narrower, cell-type specific distribution with strong enrichment in the basal ganglia (caudate, putamen, globus pallidus, and substantia nigra) in dopaminergic cells and GABAergic projections. Preclinical and human tissue studies have shown that SV2C is involved in dopaminergic transmission and is affected in Parkinson’s disease (PD).^4–6^ Studies on SV2C knockout mice and animal models of PD show decreased dopamine release and alterations to presynaptic vesicle dynamics, with impaired motor behaviors due to deficits in dopaminergic signaling.^7,8^ Studies on brain tissue from PD donors showed disruption and reduced expression of SV2C.^4^ Overall, these findings suggest that SV2C is an attractive molecular target for *in vivo* synaptic imaging with positron emission tomography (PET). We hypothesize that SV2C PET can be used as imaging tool for early detection of synaptic dysfunction and monitoring of synaptic changes in neurodegenerative disorders, particularly PD.

SV2A PET radioligands such as [^11^C]­UCB-J^9,10^ and [^18^F]­SynVesT-1 ^11^ serve as imaging markers to study synaptic density, since SV2A is uniformly and highly expressed in the brain. Across serial assessments, no significant longitudinal changes in SV2A were detected in any brain region. This indicates that SV2A PET with [^11^C]­UCB-J lacks sensitivity to progressive synaptic alterations over time, limiting its utility as a biomarker for monitoring disease progression.^12,13^ It may still be useful for cross-sectional characterization or detecting baseline group differences but not for tracking change. SV2C has a lower density than SV2A and restricted brain distribution. Therefore, the development of SV2C PET radioligands requires compound optimization of affinity for SV2C and strong selectivity over SV2A/SV2B, as well as feasibility for labeling with ^11^C or ^18^F and suitable *in vivo* pharmacokinetic properties.

[^18^F]­UCB-F is a PET radioligand for SV2C that was developed by UCB and previously evaluated in rats using autoradiography and *in vivo* PET. On autoradiography, [^18^F]­UCB-F displayed a brain distribution consistent with the expression of SV2C. However, *in vivo* PET imaging showed no evidence of specific binding of [^18^F]­UCB-F to the rat brain. The aim of this study was to further characterize [^18^F]­UCB-F as a potential SV2C PET radioligand. Specific objectives were (1) to assess the in vitro affinity and selectivity to SV2C by in vitro binding studies and *in silico* modeling of binding mode and energy of UCB-F for SV2C; (2) to radiolabel [^18^F]­UCB-F and to assess its *in vitro* binding in rat and nonhuman primate (NHP) brain tissue; (3) to assess the *in vivo* pharmacokinetic properties of [^18^F]­UCB-F in NHPs, with PET.

## Results and Discussions

### In Silico Modeling

*In-silico* modeling was used to investigate the effect of temperature on UCB-F binding to SV2C. The initial structure of SV2C was generated using homology modeling, followed by structural relaxation through MD simulations. As shown in Figure 1, UCB-F adopts a consistent and well-defined binding conformation within the SV2C binding pocket, stabilized by two key hydrogen bonds. These conserved interactions contribute to anchoring the ligand and maintaining its orientation, suggesting that hydrogen bonding may play a critical role in supporting the stability of the UCB-F–SV2C complex.

**Figure 1:**
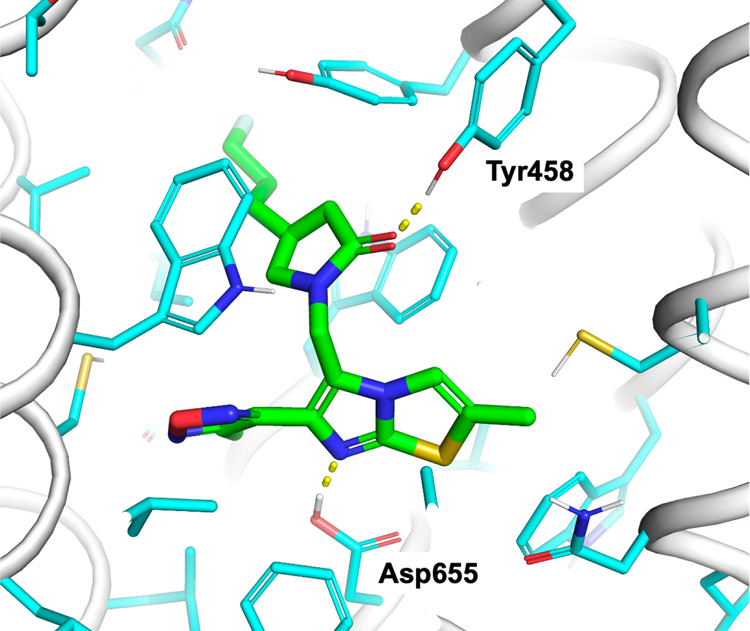
Molecular binding interactions within the SV2C–UCB-F complex. The protein backbone is shown as gray helices, and the ligand is depicted in stick representation with carbon atoms colored cyan and green, nitrogen in blue, and oxygen in red. Yellow dashed lines represent hydrogen bonds formed between the ligand and surrounding protein residues.

Molecular dynamics simulations were performed on the protein–ligand complexes at different temperatures to evaluate both the conformational stability and dynamic behavior of the predicted binding poses. The temporal evolution of ligand RMSD was monitored throughout the 100 ns simulation trajectories. Across all replicates, RMSD values consistently remained below 2.5 Å, indicating that the initial binding conformations were stably maintained over time. These results demonstrate the robustness of the predicted binding mode and confirm that UCB-F remains well anchored within the binding pocket under the simulated conditions.

While RMSD (Root Mean Square Deviation) analysis provides information on the overall structural stability of the complex, it does not capture local interactions that are important for maintaining ligand binding. In this system, hydrogen bonds between UCB-F and SV2C have been identified as key stabilizing interactions based on the predicted binding mode. Therefore, we performed additional analysis of the simulation trajectories to assess the behavior of these hydrogen bonds under different temperature conditions. One of the hydrogen bonds remained relatively stable across all temperatures, whereas the other exhibited temperature-dependent fluctuations (Figure 2). As shown in Figure 2, the lifetime of this hydrogen bond varied with temperature. At 277 K, the hydrogen bond persisted for longer periods, with a mean lifetime of 11.86 ns. As the temperature increased to 294 K and 310°K, the lifetimes decreased, with the shortest mean lifetime of 1.03 ns observed at 310°K.

**Figure 2:**
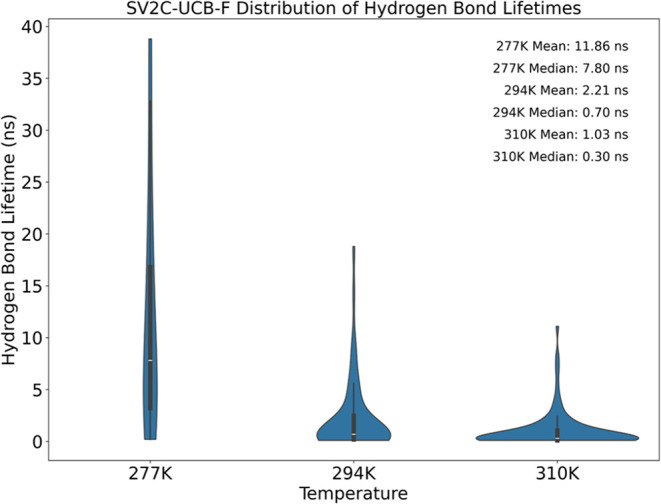
Violin plots depicting the distribution of hydrogen bond lifetimes between the UCB-F ligand and Tyr458 in SV2C. Data are shown for three different temperatures: 277 (4 °C), 294 (27 °C), and 310 K (37 °C). The distributions illustrate the stability and persistence of hydrogen bonding interactions under varying thermal conditions.

This reduction in hydrogen bond lifetime at elevated temperatures reflects a weakening of specific stabilizing interactions within the binding site, which may contribute to the lower binding affinity of UCB-F to SV2C under these conditions. Experimental data indicate that UCB-F exhibits a reduced binding affinity at higher temperatures. Therefore, our simulation results provide a structural perspective that helps to explain the experimentally observed temperature dependence of ligand binding, suggesting that decreased hydrogen-bond stability is associated with weakened binding interactions.

### In Vitro Competition Studies

In vitro competition binding (*n* = 3) demonstrates a clear temperature dependence for UCB-F, with SV2C affinity decreasing by about an order of magnitude from 277 K to 310 K, consistent with shorter hydrogen-bond lifetimes in the binding pocket at higher temperature. At 277° K, human UCB-F shows high affinity (pKi 8.4 with [^3^H]­UCB101275–1), whereas at 310 K, SV2C affinity falls to pKi ∼ 6.8–7.3 across species and radioligands (Table 1). In contrast, at 310 K, UCB-F exhibits minimal binding to human SV2A and SV2B (pKi < 6 with [^3^H]­padsevonil), establishing a robust SV2C-selective profile under physiologically relevant conditions (Table 1). Together, these data indicate that cooling artificially inflates apparent potency (pKi ∼ 8.3–8.4 at 277 K), while at 310 K UCB-F maintains selective SV2C engagement in the low- to mid–double-digit nanomolar range, with at least a 20–200× selectivity window over SV2A/SV2B depending on species and radioligand; the 277 K SV2C value is reported elsewhere.^14^

**Table 1:** In Vitro pKi (±S.E.M., *n* ≥ 3) of UCB-F to SV2 Proteins Measured at 4 °C and 37 °C^b^

radioligand	hSV2C (277 K/4 °C)	hSV2C (310 K/37 °C)	rSV2C (310 K/37 °C)	pSV2C (310 K/37 °C)	hSV2A (310 K/37 °C)	hSV2B (310 K/37 °C)
[^3^H]UCB101275–1 ^14^	8.3 ± 0.06	7.3 ± 0.21	6.9 ± 0.13	7.0 ± 0.06		
[^3^H]UCB-1A		7.4 ± 0.05	6.7 ± 0.03	6.8 ± 0.06		
[^3^H]padsevonil		6.8 ± 0.03			<5^a^	<5^a^

a There was no measurable pKi within the tested concentration range (highest concentration was 10 μM).

b [^3^H]­UCB-1A = [^3^H]­UCB4297.

### Radiolabeling of [^18^F]­UCB-F

The radiolabeling of [^18^F]­UCB-F was successfully carried out through a nucleophilic substitution process, where the respective mesylate precursor was reacted with [^18^F]­fluoride in a one-pot synthesis. This process utilized kryptofix K_2.2.2_ and potassium carbonate (K_2_CO_3_) to facilitate the reaction, as shown in Scheme 1. Several solvents, including acetonitrile, dimethylformamide (DMF), and dimethyl sulfoxide (DMSO), were evaluated at varying reaction temperatures to optimize the yield (Table 2). The combination of acetonitrile as the solvent and a reaction temperature of 100 °C maintained for 10 min proved to be the most effective, resulting in the highest incorporation yield for the synthesis.

**Scheme 1:**
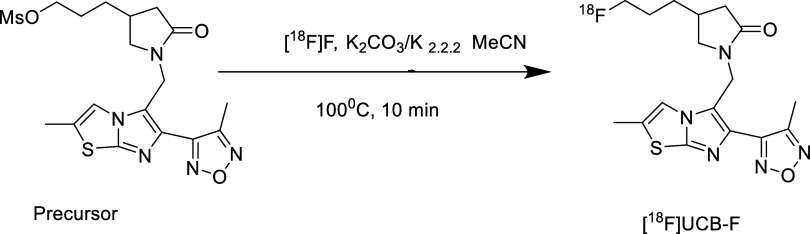


**Table 2:** Optimisation of the Radiolabeling of [^18^F]­UCB-F

amount of precursor (mg)	solvent	reaction temp (°C)	reaction time	radiochemical yield (%)
2	DMSO	70	5/15/30	<1%
		90	15/30	4–5%
		120	15/30	15%
		150	30	5%
5		100	20	10%
		125	15	5%
		150	15	1–2%
2	DMF	100	15/30	1–2%
5		120	30	5%
		150	30	0%
2	Acetonitrile	70	15	20%
		90	10/30	30%
		100	10/20/30	35%
5	Acetonitrile	100	20	35%

Following synthesis, the radiolabeled [^18^F]­UCB-F was purified using high-performance liquid chromatography (HPLC). From approximately 10 min of cyclotron irradiation at 35 μA, we obtained over 3.0 GBq of the final product. The overall radiochemical yield, nondecay corrected, was consistently above 35%. [^18^F]­UCB-F achieved radiochemical purities exceeding 99%, demonstrating the high purity of the preparations. The entire radiosynthesis workflow, including [^18^F]-fluorination, HPLC purification, solid-phase extraction (SPE) isolation, and formulation—was completed within 75 min.

### Autoradiography Studies in Rat and Nonhuman Primate Brain Tissue

[^18^F]­UCB-F binding was evaluated by autoradiography in brain sections of *n* = 3 rats and n = 2 NHP (Figure 3). The distribution of [^18^F]­UCB-F binding was comparable between corresponding brain regions in the rat and NHP and follows the expression profile of SV2C in the brain.^15–17^ The highest binding levels were observed in the brainstem, deep cerebellar nuclei (DCN), and basal ganglia, particularly within the striatum, globus pallidus, and substantia nigra (Table 3). The lowest binding was observed in cerebral and cerebellar cortex as well as the hippocampus, where immunostaining experiments have shown to express low levels of SV2C. With coincubation of 10 μM cold UCB-F, only <10% of the [^18^F]­UCB-F binding was nondisplaceable in the rat brain regions expressing high SV2C (basal ganglia, brainstem, DCN), and ∼20% in the cerebral and cerebellar cortex. In the NHP, the level of [^18^F]­UCB-F binding that was not displaced by 10 μM cold UCB-F was ∼20% in the basal ganglia and ∼40% in the cortical regions. We believe this higher level of nondisplaceable binding in the NHP sections is due to the thickness of the tissue, since the NHP sections were 20 μm thick, whereas the rat sections were 12 μm.

**Figure 3:**
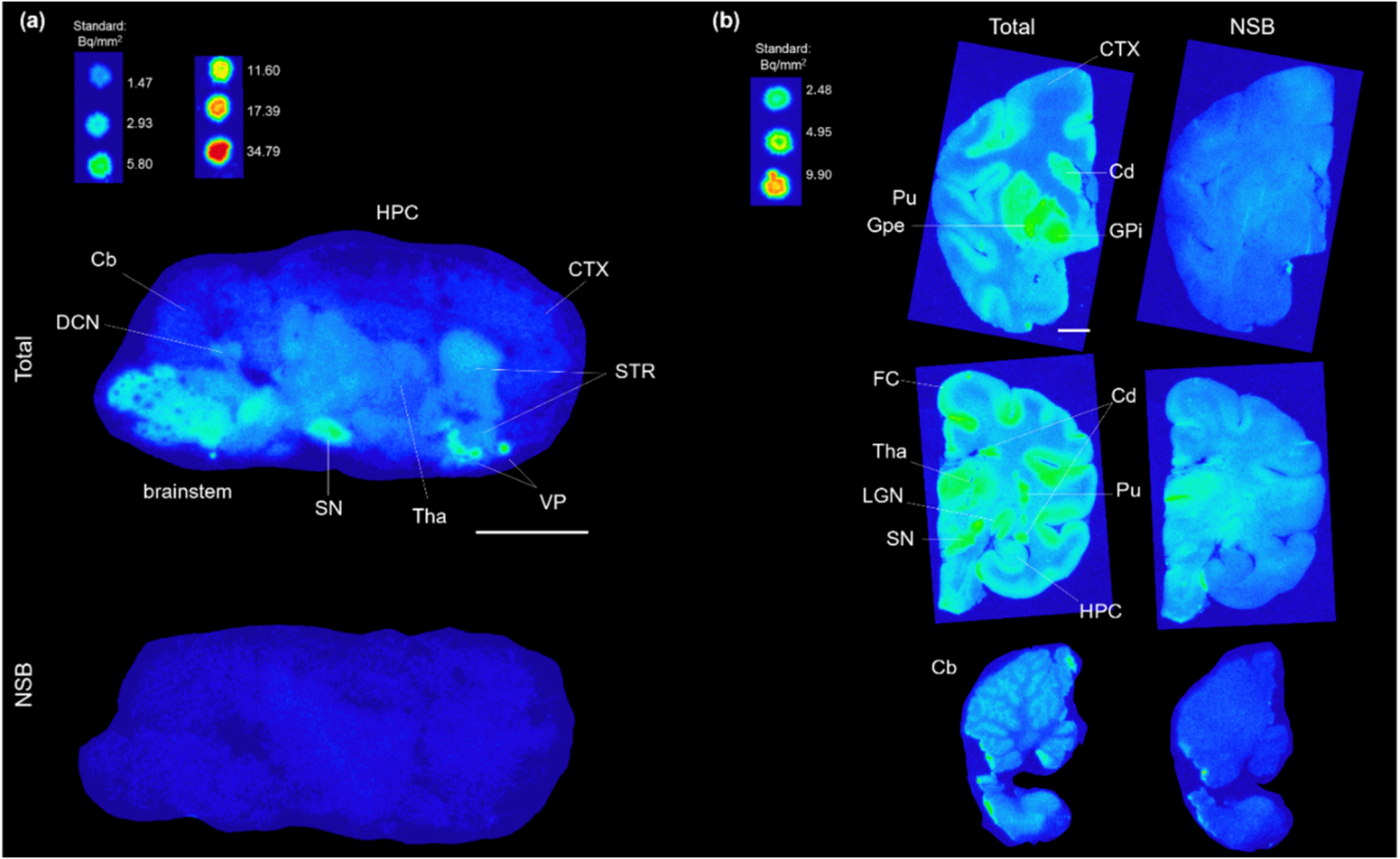
Representative autoradiograms of [^18^F]­UCB-F binding to synaptic vesicle glycoprotein 2C (SV2C). The images represent (a) rat brain sagittal sections (0.15 MBq/mL [^18^F]­UCB-F, 25.8 GBq/μmol) and (b) nonhuman primate coronal sections (0.15 MBq/mL [^18^F]­UCB-F, 15.9 GBq/μmol), alone (Total) and coincubated with excess amount (10 μM) of unlabeled UCB-F (nonspecific binding, NSB). CTX, cortex; STR, striatum; Cd, caudate nucleus; Pu, putamen; VP, ventral pallidum; GPi, internal globus pallidus; GPe, external globus pallidus; Tha, thalamus; LGN, lateral geniculate nucleus; HPC, hippocampus; SB, substantia nigra; Cb, cerebellum; DCN, deep cerebellar nuclei. Line scales: 5 mm.

**Table 3:** Specific Binding (Bq/mm^2^) of [^18^F]­UCB-F to Synaptic Vesicle Glycoprotein 2C (SV2C), Measured in Rat and Nonhuman Primate Brains^a^

	rat			non-human primate		
brain region	*n*	specific binding (Bq/mm^2^)	±SEM	*n*	specific binding (Bq/mm^2^)	±SEM
Cortex	3	0.7	0.1	2	2.6	0.2
Hippocampus	3	0.9	0.1			
Striatum	3	3.6 ^§^	0.3			
Putamen				2	4.5	0.6
Caudate nucleus				2	4.2	1.1
Ventral pallidum	3	5.2 ^§§§^	0.3			
Globus pallidus				2	5.9 *	0.6
Substantia nigra	2	5.1 ^§§§^	1.5	1	5.4	0.0
Deep cerebellar nuclei	3	2.8	0.3			
Cerebellum	3	0.9	0.1	2	0.9	0.1
Brainstem	3	4.1 ^§§^	0.1			

a Specific binding is defined as the difference between the total binding (0.15 MBq/mL [^18^F]­UCB-F) and the binding remaining in the presence of an excess amount (10 μM) of unlabeled UCB-F. Values are presented as mean specific binding (Bq/mm^2^) ± standard error of the mean (SEM) from the indicated number (*n*) of individuals. Quantification of the rat striatum consisted of the average of the putamen and caudate nucleus. ^§^ *p* < 0.05; ^§§^ *p* < 0.01; ^§§§^ *p* < 0.001, compared to rat Cortex, Hippocampus and Cerebellum; *p < 0.05, compared to NHP cerebellum, obtained via one-way ANOVA with Šídák’s multiple comparisons test.

### PET Studies with [^18^F]­UCB-F in Nonhuman Primates

Two cynomolgus monkeys, one female (NHP1) and one male (NHP2), were examined with [^18^F]­UCB-F, as detailed in Table 4. The injected radioactivity of [^18^F]­UCB-F was measured at 160 MBq and 144 MBq, with an injected mass of 2.3 and 3.4 μg. Summated PET images for the two baseline studies are presented in Figure 4. Under baseline condition, [^18^F]­UCB-F showed whole-brain uptake peaking at 3.4 SUV for NHP1 and 3.0 SUV for NHP2 (Figure 5). An initial rapid increase in radioligand uptake was observed across the entire brain, with minimal SUV variation among regions (Figure 6). The radioligand exhibited rapid washout across all regions, indicating reversible kinetics.

**Figure 4:**
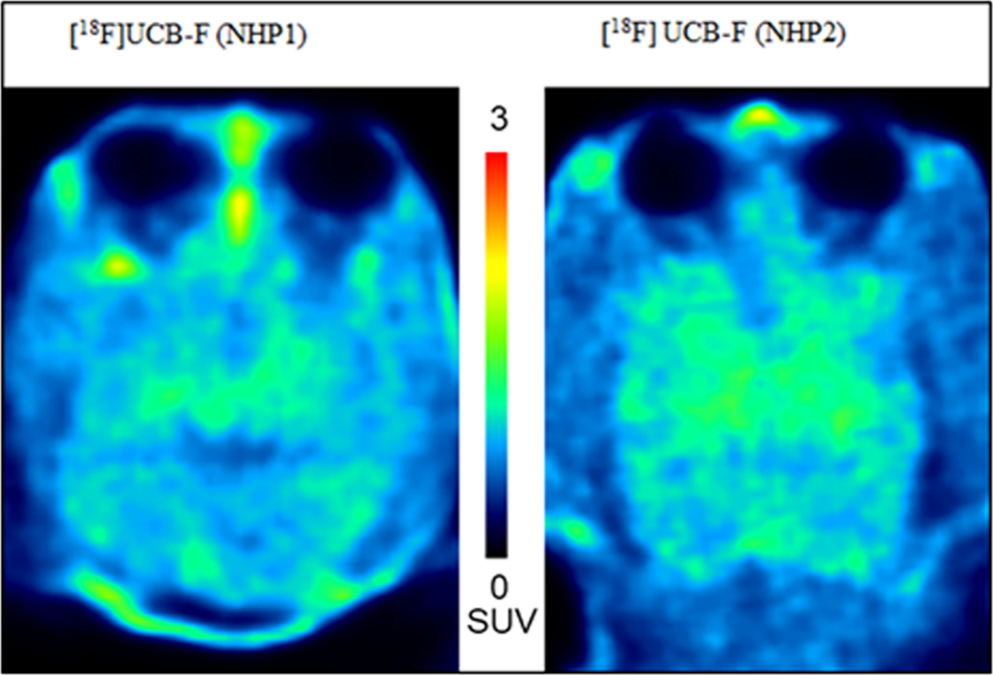
PET images in NHP 1 and NHP 2 after administration of [^18^F]­UCB-F.

**Figure 5:**
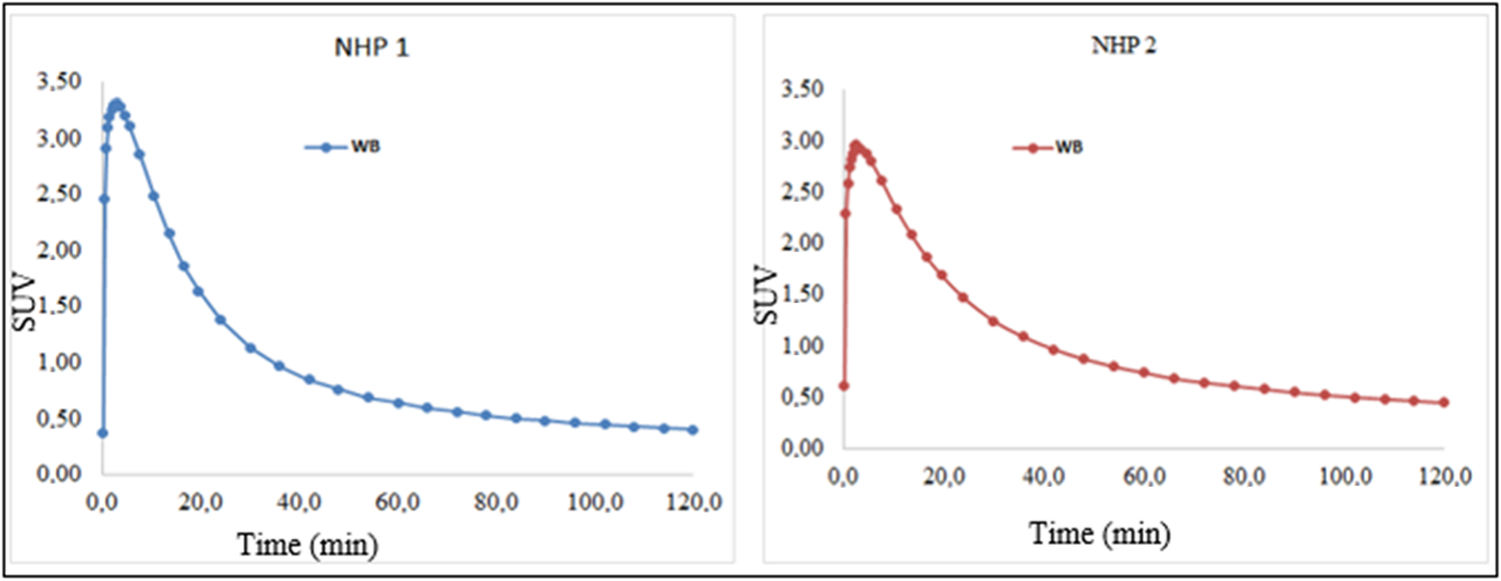
Time activity curves representing whole brain (WB) uptake of [^18^F]­UCB-F in NHP 1 and in NHP 2 at baseline condition.

**Figure 6:**
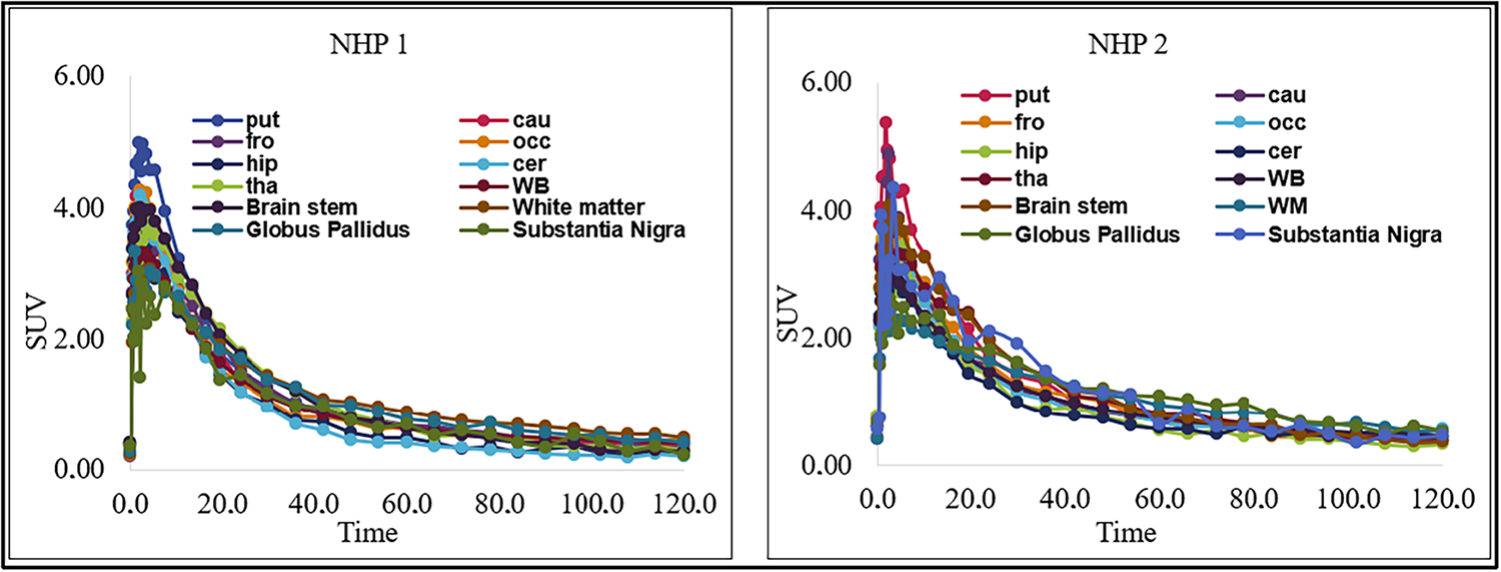
Time activity curves representing regional brain uptake of [^18^F]­UCB-F in NHP 1 and in NHP 2 at baseline condition.

**Table 4:** Body weight of NHPs, Injected Radioactivity, Mass, and the MA at the Time of Administration

NHP ID	BW (g)	injected radioactivity (MBq)	molar activity (GBq/μmol)	injected mass (μg)
NHP 1	8100	160	26	2.3
NHP 2	1100	144	16	3.4

### Radiometabolite Analysis

After deproteinization, over 95% of the plasma radioactivity was effectively recovered in acetonitrile. HPLC analysis following the injection of [^18^F]­UCB-F into plasma showed the compound eluting at a retention time of 6.5 min (Figure 7A). Initially, the parent compound was most abundant at 2 min, accounting for approximately 92%, but this decreased to around 22% at 45 min during PET studies, as reflected in the percentage of unchanged radioligand across both PET experiments (Figure 7B). Additionally, several more polar radiometabolite peaks appeared before the parent peak during the elution. The identity of [^18^F]­UCB-F was confirmed through coinjection with its nonradioactive counterpart. Radiometabolite analyses indicate rapid plasma metabolism of [^18^F]­UCB-F with a low parent fraction at later time points. This likely reduces effective tracer delivery to the brain, shortens the usable imaging window, and increases noise/bias in metabolite-corrected input functions, compounding the reduced affinity observed at physiological temperature. Potential brain-penetrant radiometabolites cannot be excluded, and even early time analyses are constrained by fast clearance.

**Figure 7:**
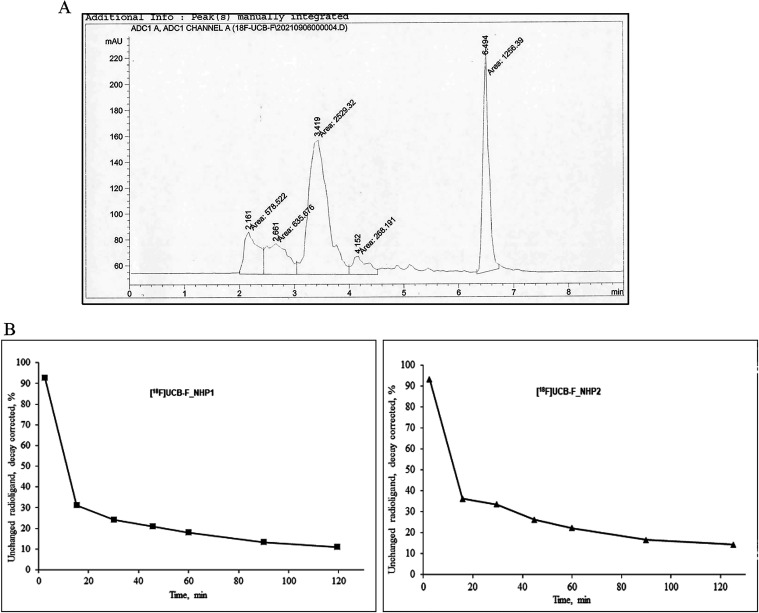
*In vivo* metabolism of [^18^F]­UCB-F is shown as the relative plasma composition of the parent compound at 30 min time point of NHP1. The *in vivo* metabolism of [^18^F]­UCB-F is shown as the relative plasma composition at baseline condition in NHP1 and NHP2.

## Conclusion

[^18^F]­UCB-F was successfully radiolabeled with high incorporation yield. *In vitro*, it showed specific binding on tissue slices from rodent and NHP brains. However, *in vivo*, [^18^F]­UCB-F displayed rapid washout from the brain, lack of regional brain distribution, and rapid metabolism. The discrepancy between autoradiography and *in vivo* PET studies is likely explained by the decrease in the affinity of [^18^F]­UCB-F to SV2C that was observed at 37 °C compared with the affinity measured at 4 °C. *In silico* modeling studies suggested that the effect of temperature on the affinity of UCB-F to SV2C can be explained by the decreased hydrogen bond stability of the binding pose. Overall, these findings indicate that [^18^F]­UCB-F is not a suitable PET radioligand for imaging SV2C and additional work is ongoing to identify a potential candidate.

## Materials and Methods

### *In Silico* Calculations Including Machine Learning

The initial structure of SV2A was obtained from the Protein Data Bank using PDB ID: 8UO9 ^18^ and prepared using the Protein Preparation Wizard in Schrödinger. The UCB-F ligand was positioned in the binding site by aligning it with the cocrystallized reference ligand, UCB-2500. The OPM (Orientations of Proteins in Membranes) Web server^19^ was used to determine the appropriate orientation of the protein within a lipid bilayer.

The SV2C–UCB-F complex was generated via homology modeling using the SV2A–UCB-F structure as a template. System construction for SV2C was carried out using Schrödinger’s System Builder module. The complex was embedded in a POPC lipid bilayer, solvated with TIP3P water in an orthorhombic box with a 10 Å buffer between the protein and the box edges, and neutralized with Na^+^ and Cl^–^ ions to achieve a physiological salt concentration of 0.15 M. A 10 ns equilibration simulation was performed to relax the system prior to production runs.

Production molecular dynamics simulations were conducted at three temperatures (277, 294, and 310 K) to investigate temperature-dependent effects on binding dynamics. All simulations were performed using the OPLS4 force field implemented in Desmond (Schrödinger 2023–2).^20^ Production runs were extended to 100 ns to ensure adequate conformational sampling. For each temperature condition, three independent replicates were performed with different initial random seeds. Trajectory analyses were conducted using Desmond’s built-in tools, complemented by custom Python scripts.

### Radiochemistry

#### General

The nonradioactive reference standard UCB-F (4-(3-(fluoropropyl)-1-((2-methyl-6-(4-methyl-1,2,5-oxadiazol-3-yl)­imidazo­[2,1-*b*]­thiazol-5-yl)­methyl)­pyrrolidin-2-one), as well as the corresponding precursor 3-(1-((2-methyl-6-(4-methyl-1,2,5-oxadiazol-3-yl)­imidazo­[2,1-*b*]­thiazol-5-yl)­methyl)-5-oxopyrrolidin-3-yl)­propyl methanesulfonate—were synthesized by Advinus Therapeutics, Bangalore-560058, India. All other chemicals and reagents were sourced from commercial suppliers and used without further purification. Solid-phase extraction (SPE) cartridges, specifically SepPak QMA Light and SepPak C18 Plus, were purchased from Waters, Milford, Massachusetts, USA. Before use, the C18 Plus cartridges were activated by rinsing with 10 mL of ethanol followed by 10 mL of sterile water. The SepPak QMA Light cartridges were activated using 10 mL of 0.5 M K_2_CO_3_ solution, followed by rinsing with 15 mL of 18 MΩ water.

Fluorine-18 fluoride ([^18^F]­F) was produced at Karolinska Hospital in Stockholm, Sweden, using a 16.4 MeV GEMS PET trace cyclotron (GE, Uppsala, Sweden). Radiolabeling was performed using a custom-made semiautomated or automated synthesis module. Liquid chromatography (LC) analysis was carried out on a Merck-Hitachi gradient pump with a Merck-Hitachi L-4000 variable-wavelength UV detector. LC-MS analyses were performed using either a Waters Quattro Premier micro mass spectrometer or a Waters SQD 3001 single quadrupole mass spectrometer, both connected to Waters Acquity UPLC systems.

#### Production of [^18^F]­Fluoride ([^18^F]­F^–^)

The production of ^18^F-/K_2_CO_3_/Kryptofix_2.2.2_ complex, an organic cation/inorganic anion salt, was carried out following a previously published method.^21^ To generate [^18^F]­F–, a GEMS PETtrace cyclotron was used to irradiate ^18^O-enriched water with 16.4 MeV protons via the ^18^O­(p,n)^18^F reaction. The produced [^18^F]­F– was separated from the ^18^O–H2O using a conditioned SepPak QMA Light anion exchange cartridge, which was then washed with a solution containing potassium carbonate (13 μmol, 1.8 mg) and kryptofix 2.2.2 (26 μmol, 9.8 mg) in 85 μL of 18 MΩ water and 2 mL of acetonitrile (MeCN). This mixture was transferred into a reaction vessel (either 10 or 4 mL). The solvents were evaporated at 140 °C under a continuous flow of nitrogen/helium (70 mL/min) for 10 min, resulting in a dry complex of [^18^F]­F–/K_2_CO_3_/K_2.2.2_. After evaporation, the residue was cooled to room temperature.

#### Synthesis of [^18^F]­UCB-F (4-(3-[^18^F]­fluoropropyl)-1-((2-methyl-6-(4-methyl-1,2,5-oxadiazol-3-yl)­imidazo­[2,1-*b*]­thiazol-5-yl)­methyl)­pyrrolidin-2-one)

To the dry complex of ^18^F–F-/K_2_CO_3_/K_2.2.2_., corresponding mesylate-precursor (1 mg, 0.005 mmol) in acetonitrile (500 μL) was added at 100 °C and left for 10 min to produce [ ^18^ F]­UCB-F. The reaction mixture was cooled to room temperature (RT) and diluted with water (2.5 mL) to a total volume of 3 mL before being injected into a semipreparative reverse-phase XBridge HPLC column (C18, 10 mm × 250 mm, 5 μm) for purification. The column outlet was connected to a UV absorbance detector (λ = 254 nm) in series with a GM-tube for radioactivity detection. Elution was carried out with a mobile phase consisting of CH_3_CN and ammonium formate (0.1M) in a ratio of 24:76 at a flow rate of 6 mL/min. This process yielded a radioactive fraction corresponding to pure [^18^F]­1/[^18^F]­2, with a retention time (*t*_R_) of 20–22 min and collected in a bottle containing sterile water (50 mL). The resulting mixture was further purified by passing through a preconditioned SepPak SPE (Oasis HLB 3 cm^3^) cartridge followed by washing with sterile water (10 mL). Isolated [^18^F]­1/[^18^F]­2 was eluted with 1 mL of ethanol into a sterile vial containing 9 mL of sterile saline. The formulated product was then filtered through a Millipore Millex GV sterile filter unit (0.22 μm) for further application in PET.

##### *In Vitro* Autoradiography

Sectioning of the rat and NHP tissue and autoradiography assays were performed, as previously described,^22^ at the Autoradiography Core Facility, Department of Clinical Neuroscience, Division of Imaging Core Facilities and Centre for Imaging Research (CIR), at the Karolinska Institutet.

Studies using NHP brain tissue were approved by the Animal Ethics Committee of the Swedish Animal Welfare Agency (registration no. 4820/06–600 and 399/08). Sections from the brains of 2 adult female cynomolgus monkeys (Macaca Fascicularis) were used in this study. Coronal sections from the snap-frozen brains of *n* = 3 adult naïve Sprague–Dawley rats were sectioned at 12 μm thickness, and NHP Brains were cut at 20 μm using a cryomicrotome (CM1860, Leica Biosystems, Nußloch, Germany), thaw-mounted onto glass slides, and stored at −20 °C until the autoradiography assay was performed. For the [^18^F]­UCB-F binding autoradiography assay, all sections were first preincubated for 20 min at room temperature with binding buffer (50 mM Tris HCl, pH 7.4, containing 120 mM NaCl, 5 mM KCl, 2 mM CaCl2, 1 mM MgCl2), then incubated at room temperature with [^18^F]­UCB-F (0.15 MBq/mL) for 55 min. The rat and NHP autoradiography assays were performed in separate days. The molar activity measured was 25.8 GBq/μmol in the rat autoradiography assay and 15.9 GBq/μmol in the NHP autoradiography assay. Nonspecific binding was determined by coincubation with 10 μM of unlabeled UCB-F. After incubation, sections were washed 3 × 5 min at 4 °C, in 50 mM Tris HCl, pH 7.4, briefly dipped in ice-cold distilled water, then dried in a heat plate before being exposed to storage phosphor screens (BAS-IP SR2025, Fujifilm, Tokyo, Japan) overnight.

Radioactivity was detected and quantified with an Amersham Typhoon FLA-9500 phosphor imaging scanner (Cytiva, Marlborough, MA, USA). Autoradiograms were analyzed using Multi Gauge 3.2 phosphor imager software (Fujifilm, Tokyo, Japan). The measured photostimulated luminescence (PSL)/mm^2^ values were converted into decay-corrected radioactivity units based on the signal intensity from standard quantities (1.5 kBq to 2.9 kBq), diluted from the respective [^18^F]­UCB-F batch, and pipetted (20 μL per standard) onto filter papers exposed in each storage phosphor plate. Binding values from two replicates of total and nonspecific conditions from each individual were plotted using GraphPad Prism v10 (GraphPad Software, Boston, MA, USA) and specific binding was determined by subtracting the binding signal in the presence of 10 μM unlabeled (cold) UCB-F from the total binding.

### Study Design in Nonhuman Primates (NHP), PET Procedure, and Quantification

This research study adhered to ethical standards and received approval from the Animal Ethics Committee in Stockholm, administered by the Swedish Board of Agriculture (10367–2019). The study also complied with the guidelines outlined in ″Guidelines for Planning, Conducting, and Documenting Experimental Research″ (Dnr 4820/06–600) of Karolinska Institutet. The nonhuman primates (NHPs) used in this study were housed at the Astrid Fagraeus Laboratory, Comparative Medicine, in Solna, Sweden. Two male cynomolgus NHPs were selected for participation. The study involved performing brain PET scans on both NHPs under the baseline conditions.

At the Astrid Fagraeus Laboratory, each nonhuman primate (NHP) received ketamine hydrochloride at 10 mg kg-1 by intramuscular injection to induce anesthesia. An inflatable cuff was inflated on the delivery hose until capnography confirmed clear alveolar waves. Endotracheal intubation-maintained anesthesia was achieved with a blend of sevoflurane, oxygen, and medical air. A three-point head holder fixed the skull, while an esophageal probe fed a Bair Hugger 505 unit (Arizant Healthcare, MN) to provide warmth. Heart rate, blood pressure, respiratory rate, and SpO2 were recorded on a bedside monitor, and saline was dripped continuously to correct volume losses.

Positron emission tomography was performed on a LFER PET-CT system (Mediso).^23^ [^11^C]­UCB-1A was administered as bolus, followed by the administration of 2 mL NaCl. PET data were acquired in list mode for 123 min and reconstructed with a series of frames of increasing duration (9 × 20 s, 3 × 60 s, 5 × 180 s, 17 × 360 s). The reconstruction was performed using the Tera-Tomo 3D algorithm (with 10 iterations and 9 subsets), including detector modeling with attenuation and scatter correction based on a 3-component material map. Volumes of interest (VOIs) were delineated on an MR image of the animal, which was manually coregistered with the average PET image using the FUSION tool in the PMOD software (version 4.2; Bruker). Selected VOIs were the caudate, putamen, globus pallidus, substantia nigra, and cerebellum. The radioactivity concentration was expressed as SUV. Venous blood samples were collected at 2, 15, 30, 45, 60, 90, and 120 min for radioactivity measurements and radiometabolite analyses.

Magnetic resonance imaging was performed on a General Electric DISCOVERY MR750 3.0 T scanner (Milwaukee, WI, USA). A T1-weighted sequence was acquired to facilitate coregistration with PET and delineate cortical and subcortical regions. Data analysis began with the investigator hand-drawing regions of interest (ROIs) on the MRIs of each nonhuman primate, outlining the entire brain as well as the cerebellum, caudate, putamen, thalamus, frontal cortex, occipital cortex, and hippocampus. Next, the PET scans covering the full imaging session were precisely aligned to each animal’s structural MRI. The alignment parameters were then transferred to the dynamic PET data set, allowing time-activity curves to be extracted from all specified brain regions. Finally, the average standardized uptake value (SUV) was computed for each delineated ROI.

### Radiometabolite Analysis

Radiometabolites present in the blood plasma of nonhuman primates (NHPs) were analyzed using a previously published method.^24^ Throughout the PET imaging session, the proportions of radioactivity attributable to [^18^F]­UCB-F and its radioactive metabolites in NHP plasma were quantified by using a reverse-phase high-performance liquid chromatography (HPLC) system. This system employed a UV detector set at 254 nm in conjunction with a radioactivity detector.

Arterial blood samples, ranging from 1.0 to 3.0 mL, were manually collected at various time points, including 2.5, 15, 30, 45, 60, 90, and 120 min after injection of [^18^F]­UCB-F. Postcollection, blood samples were centrifuged at 4000 rpm for 2 min to isolate plasma, which was then diluted 1.4-fold with acetonitrile and centrifuged at 6000 rpm for 4 min. The supernatant was separated from the pellet and further diluted with 3 mL of water. For the separation of radiometabolites from the unchanged [^18^F]­UCB-F, a high-performance liquid chromatography (HPLC) system was employed. The setup included an Agilent 1200 series binary pump connected to a Rheodyne 7725i manual injection valve with a 5.0 mL loop, and a radiation detector (Oyokoken S-2493Z) enclosed within a 50 mm lead shield. A semipreparative ACE 5 μm C18 HL column (250 × 10 mm) was used for chromatographic separation, utilizing gradient elution. The mobile phase consisted of acetonitrile (A) and 0.1 M ammonium formate (B), flowing at 5.0 mL/min. The gradient program involved: 0–7.0 min, changing from 40:60 to 90:10 (A/B) v/v; 7.0–9.0 min, maintaining at 90:10; and 9.0–9.1 min, returning from 90:10 to 40:60 (A/B) v/v. The radioactive peaks were integrated, with the peak corresponding to [^18^F]­UCB-F expressed as a percentage of the total detected radioactivity. To assess the system’s recovery rate, a 2 mL aliquot of the collected eluate was measured and divided by the total radioactivity.

## Data Availability

All the supporting data are stored at the Karolinska Institutet archive.
